# Perioperative blood transfusion has distinct postsurgical oncologic impact on patients with different stage of hepatocellular carcinoma

**DOI:** 10.1186/s12885-020-06980-5

**Published:** 2020-05-29

**Authors:** Gui-Xing Chen, Chao-Ying Qi, Wen-Jie Hu, Xiao-Hui Wang, Yun-Peng Hua, Ming Kuang, Bao-Gang Peng, Shao-Qiang Li

**Affiliations:** 1 Department of Liver Surgery, The First Affiliated Hospital of Sun Yat-sen University, No. 58 Zhongshan Er Road, Guangzhou, 510080 China; 2 Department of Operating Center, The First Affiliated Hospital of Sun Yat-sen University, No. 58 Zhongshan Er Road, Guangzhou, 510080 China

**Keywords:** Hepatocellular carcinoma, Blood transfusion, Outcomes, Hepatectomy

## Abstract

**Background:**

The influence of perioperative blood transfusion (PBT) on postsurgical survival of patients with different stage of hepatocellular carcinoma (HCC) is not well clarified. This study aimed to evaluate the impact of PBT on survival outcomes of different stage of HCC patients.

**Methods:**

Consecutive patients who underwent liver resection for HCC between January 2009 and November 2015 were identified from an HCC prospective database in authors’ center. The survival outcomes were compared between patients receiving PBT and those without PBT before and after propensity score matching (PSM) in different stage subsets. Cox regression analysis was performed to verify the impact of PBT on outcomes of HCC.

**Results:**

Among 1255 patients included, 804 (64.1%) were Barcelona Clinic Liver Cancer (BCLC) stage 0-A, and 347 (27.6%) received PBT. Before PSM, patients with PBT had worse disease free survival (DFS) and overall survival (OS) compared with those without PBT in both BCLC 0-A subset and BCLC B-C subset (all *P* < 0.05). After PSM, 288 pairs of patients (with and without PBT) were created. In the subset of BCLC 0-A, the median DFS of patients with PBT was shorter than those without PBT (12.0 months vs. 36.0 months, *P* = 0.001) Similar result was observed for OS (36.0 months vs. 96.0 months, *P* = 0.001). In the subset of BCLC B-C, both DFS and OS were comparable between patients with PBT and those without PBT. Cox regression analysis showed that PBT involved an increasing risk of DFS (HR = 1.607; *P* < 0.001) and OS (HR = 1.756; *P* < 0.001) for this subset. However, PBT had no impact on DFS (*P* = 0.126) or OS (*P* = 0.139) for those with stage B-C HCC.

**Conclusions:**

PBT negatively influenced oncologic outcomes of patient with BCLC stage 0-A HCC, but not those with stage B-C after curative resection.

## Background

Hepatocellular carcinoma (HCC) is the fifth most common tumor worldwide, and it is the second leading cause of cancer-related death in China [1]. Liver resection is the mainstay curative treatment for early-stage HCC and selected intermediate-stage or advanced-stage HCC with preserved liver function [2]. As the resection technique and perioperative management have improved, surgical morbidity and mortality following hepatectomy have substantially decreased [3, 4]. In particular, refined surgical manipulation involves reduced blood loss during liver resection; however, liver resection for HCC involves a high risk of bleeding due to underlying cirrhosis. The blood transfusion rate during liver resection has decreased from 66 to 22% in the past two decades [5].

Blood transfusion is still a life-saving therapy when excessive intraoperative bleeding occurs, but it involves the risk of transfusion-related complications, such as transmission of hepatitis viruses, human immunodeficiency virus, and allergic reactions [6]. Regarding oncologic outcomes, although many studies had been reported, the influence of perioperative blood transfusion (PBT) on postoperative survival outcomes is controversial [7–12]. Furthermore, the influence of PBT on different stage of resectable HCC has not been well investigated.

In this study, we focused on the impact of PBT on oncologic outcomes of patient with different stage of HCC after curative resection by using propensity score matching (PSM) analysis and Cox regression analysis.

## Methods

### Patients

From January 2009 to November 2015, all consecutive patients with HCC undergoing curative liver resection (complete resection of gross tumors with a pathological tumor free margin) in the authors’ department were evaluated for this study. Clinical data were entered prospectively in an HCC database and reviewed retrospectively. Patients with HCC with bile duct tumor thrombus or ruptured HCC treated with hepatectomy, those who died within 30 days postoperatively (surgical mortality) were excluded. This study was approved by the ethics committee of The First Affiliated Hospital of Sun Yat-sen University, and written informed consent was obtained from all patients.

### Perioperative assessment

Preoperative evaluation of and resection criteria for HCC at our center were previously described [13]. The treatment option was decided by the HCC multidisciplinary team. The Barcelona Clinic Liver Cancer (BCLC) staging system was used for HCC staging [14]. Although we used the Child-Pugh score to evaluate liver function in clinical practice in this cohort of patients, we used albumin to bilirubin (ALBI) scores for data analysis because it was reported that they are more accurate and objective than conventional Child-Pugh scores [15, 16]. The neutrophil-to-lymphocyte ratio (NLR) was obtained by dividing the neutrophil count by the lymphocyte count. The platelet-to-lymphocyte ratio (PLR) referred to the platelet count subtracted from the lymphocyte count. The alanine transaminase (ALT)-to-platelet ratio index (APRI) was calculated as follows: [ALT ÷ (upper limit of ALT × platelet count)] × 100. These inflammatory parameters were transformed to binary variables in the Cox regression analysis by using their median values as the cutoff thresholds, respectively.

PBT referred to the transfusion of packed red blood cells (RBCs) during excessive intraoperative bleeding or postoperative bleeding complications. Transfusions of platelets, fresh-frozen plasma, and albumin were not included. The PBT criteria were preoperative anemia (hemoglobin ≤70 g/L) and excessive intraoperative or postoperative intra-abdominal bleeding with unstable hemodynamics or hemoglobin < 70 g/L. Postoperative complications were graded by the Clavien-Dindo classification [17].

### Surgical procedures

Liver resection included anatomical resection (AR) and non-anatomical resection (NAR), which was introduced in our previous report [13]. Briefly, AR was planned for central tumors, tumors with ipsilateral satellite nodules, or portal vein tumor thrombus (PVTT), and for patients with a sufficient liver remnant after AR. NAR was preferred for peripheral tumors and for patients with an insufficient liver remnant after AR was performed. The Pringle maneuver was applied if necessary. Major resection was defined as resection larger than three segments.

### Propensity score matching analysis

To minimize the influence of patient selection bias and confounding variables between groups in this retrospective study, a PSM analysis was used [18, 19]. In this study, four levels of outcome-related variables, including patient and underlying liver disease-related [age, sex, preoperative hemoglobin level, platelet count, positive HBsAg, cirrhosis, prothrombin time (PT), alanine transaminase (ALT) level, ALBI grade], tumor-related [tumor size, tumor number, tumor capsule, microvascular invasion (MVI), portal vein tumor thrombus (PVTT), hepatic vein tumor thrombus (HVTT), alpha fetoprotein (AFP) level, tumor differentiation], systemic inflammation – related (NLR, PLR, APRI), and procedure-related variables (extent of resection, resection type, resection margin, and Pringle maneuver), were included in the propensity score model to balance the baseline of groups as much as possible. PSM was performed using R software (R 2.15.3; http://www.r-project.org). A one-to-one nearest neighbor matching without replacement algorithm was applied. To obtain the best trade-off between homogeneity and retained sample size, caliper widths of 0.20, 0.10, 0.050, and 0.010 were tested in our cohort of patients. We found that a caliper width of 0.1 met the requirement.

### Follow-up

The follow-up protocols for HCC and treatment of recurrent HCC at our center were described previously [13]. The main outcomes of this study were disease free survival (DFS) and overall survival (OS). DFS was calculated from the date of tumor resection to the date of first tumor recurrence or the last follow-up visit. The OS was calculated from the date of tumor resection to the date of death or the date of the last follow-up visit. The endpoint follow-up was December 30, 2016. The median follow-up period was 51.0 months (range, 3–102 months). The treatments of recurrent HCC including radiofrequency ablation, re-hepatectomy, transarterial chemoembolization, or sorafenib alone or combined therapy based on the number, location of recurrent tumor and liver function reserve.

### Statistical analysis

The clinical database was established using SPSS for Windows (version 22.0; IBM, Armonk, NY, USA). Continuous data are expressed as mean (standard deviation) or median (range). The independent *t* test or Mann-Whitney U test was used to compare continuous data between groups, and the χ^2^ test was used for discrete data. Cumulative DFS and OS rates were calculated using the Kaplan–Meier method and compared between groups using the log rank test. A Cox regression model involving univariable and multivariable analyses was used to identify risk factors associated with DFS and OS. All factors with statistical significance (*P* < 0.05) in the univariable analysis were entered into the multivariable analysis (forward method) to yield independent risk factors. *P* < 0.05 was considered statistically significant.

## Results

A total of 1336 patients had surgery for HCC in this study period. Eighty-one patients were excluded from this study: 17 patients with bile duct tumor thrombus; 53 patients with rupture HCC; and 11 (0.9%, 11/1266) patients who died of postoperative liver failure. Finally, 1255 patients who underwent liver resection with curative intent were recruited in this study. Most patients (84.8%) had underlying HBV infection. 27.6% (347/1255) received PBT. The patients were classified into two subsets: the BCLC 0-A subset (*n* = 804, 64.1%) and the BCLC B-C subset (*n* = 451, 35.9%) according to tumor stage.

### Patients’ clinicopathologic features in the entire cohort

The baseline clinical data of patients with PBT and those without PBT (non-PBT) within the BCLC 0-A subset and the BCLC B-C subset were compared respectively and summarized in Table 1. Numerous variables were significantly different between patients with PBT and those without PBT within each subset. 21.3% (171/804) of patients received PBT in the subset of BCLC 0-A, and 39.0% (176/451) in the BCLC B-C subset.

**Table 1:** Baseline characteristics of patients with PBT and those without PBT in different HCC stage subset in the entire cohort (*n* = 1255)

*Variable*	BCLC 0-A (*n* = 804)			BCLC B-C (*n* = 451)		
	PBT *n* = 171)	Non-PBT (*n* = 633)	*P*-value	PBT (*n* = 176)	Non-PBT (*n* = 275)	*P*-value
*Demographic factors*						
Age, yr	52.9 ± 12.5	50.9 ± 12.1	0.062	49.9 ± 12.2	49.1 ± 12.5	0.544
Sex, n (%)						
Male	139 (81.3)	557 (88.0)	0.220	157 (89.2)	249 (90.5)	0.644
Female	32 (18.7)	76 (12.0)		19 (10.8)	26 (9.5)	
HBsAg, n (%)						
Positive	143 (83.6)	545 (86.1)	0.415	148 (84.1)	228 (82.9)	0.743
Negative	28 (16.4)	88 (13.9)		28 (15.9)	47 (17.1)	
Cirrhosis, n (%)						
Yes	123 (71.9)	427 (67.5)	0.265	133 (75.6)	198 (72)	0.404
No	48 (28.1)	206 (32.5)		43 (24.4)	77 (28)	
Hemoglobin, g/L	129.7 ± 22.8	141.6 ± 17.5	< 0.001	131.2 ± 23.1	140.3 ± 19.9	0.000
Platelet count, × 10^9^ /L	185.0 ± 64.5	201.3 ± 92.9	0.008	215.4 ± 113.3	207.1 ± 71.3	0.338
Prothrombin time, s	13.0 ± 1.6	12.7 ± 0.9	0.001	13.1 ± 1.1	12.7 ± 1.3	0.001
ALT, U/L, median (range)	39 (6565)	33 (71428)	0.024	42.5(8237)	38.0(6522)	0.189
ALBI grade, n (%)						
Grade 1	84 (49.1)	399 (63.0)	< 0.001	76 (43.2)	160 (58.2)	0.001
Grade 2	84 (49.1)	232 (36.7)		99 (56.3)	115 (41.8)	
Grade 3	3 (1.8)	2 (0.3)		1 (0.5)	0	
*Inflammatory factors*						
NLR, median (range)	2.4 (0.5,13.0)	1.9 (0.3,24.9)	< 0.001	2.5 (0.9,24.4)	2.3 (0.6,18.3)	0.013
PLR, median (range)	121.1 (21.61432.1)	96.9 (19.4414.0)	< 0.001	133.9 (19.8751.0)	119.6 (20.6314.6)	< 0.001
APRI, median (range)	0.6 (0.1,12.2)	0.5 (0.1,21.1)	0.038	0.6 (0.1,3.8)	0.5 (0.1,5.7)	0.079
*Tumor characteristics*						
AFP, ug/L						
≥ 400	99 (42.1)	214 (33.8)	0.044	88 (50)	149 (54.2)	0.387
< 400	72 (57.9)	419 (66.2)		88 (50)	126 (45.8)	
Tumor size, cm	9.2 ± 6.1	6.2 ± 3.1	< 0.001	10.7 ± 3.92	8.9 ± 3.5	< 0.001
Tumor number, n (%)						
Solitary	167 (97.7)	596 (94.2)	0.049	59 (33.5)	87 (31.6)	0.975
2	4 (2.3)	29 (4.6)		59 (33.5)	102 (37.1)	
3	0 (0)	8 (1.2)		18 (10.2)	25 (9.1)	
4	0 (0)	0 (0)		40 (22.8)	61 (22.2)	
Tumor capsule, n (%)						
Complete	137 (80.1)	574 (90.7)	< 0.001	107 (60.8)	187 (68)	0.118
Incomplete	34 (19.9)	59 (9.3)		69 (39.2)	88 (32)	
Differentiation, n (%)						
I+ II	115 (67.3)	465 (73.5)	0.108	119 (67.6)	193 (70.2)	0.566
III, IV	56 (32.7)	168 (26.5)		57 (32.4)	82 (29.8)	
MVI, n (%)						
Yes	50 (29.2)	123 (19.4)	0.006	79 (44.9)	105 (38.2)	0.158
No	121 (70.8)	510 (80.6)		97 (55.1)	170 (61.8)	
PVTT, n (%)						
Yes	0	0		100 (56.8)	116 (42.2)	0.002
No	171 (100)	633 (100)		76 (43.2)	159 (57.8)	
HVTT, n (%)						0.000
Yes	0	0		21 (11.9)	7 (2.5)	
No	171 (100)	633 (100)		155 (88.1)	268 (97.5)	
*Surgical factors*						
Extent of resection, n (%)						
Major	64 (37.4)	194 (30.6)	0.092	126 (71.6)	180 (65.5)	0.174
Minor	107 (62.6)	439 (69.4)		50 (28.4)	95 (34.5)	
Type of resection, n (%)						
Anatomical	62 (36.3)	203 (32.1)	0.302	97 (55.1)	150 (54.5)	0.906
Nonanatomical	109 (63.7)	430 (67.9)		79 (44.9)	125 (45.5)	
Resection margin						
≤ 1 cm	32 (18.7)	58 (9.2)	< 0.001	56 (31.8)	122 (44.4)	0.008
> 1 cm	139 (81.3)	575 (90.8)		120 (68.2)	153 (55.6)	
Pringle maneuver, n (%)						
Yes	111 (64.9)	380 (60.0)	0.245	114 (64.8)	169 (61.4)	0.477
No	60 (35.1)	253 (40.0)		62 (35.2)	106 (38.6)	
Blood loss, ml, median (range)	1484.7 (200,12,000)	200 (50,3000)	< 0.001	1000 (200,10,500)	300 (30,2500)	< 0.001
Clavien-Dindo grade						
I	4 (2.3)	9 (1.4)	0.045	3 (1.7)	8 (2.9)	0.950
II	1 (0.6)	9 (1.4)		2 (1.1)	4 (1.5)	
III	15 (8.8)	31 (4.9)		10 (5.7)	18 (6.5)	
IV	2 (1.2)	3 (0.5)		3 (1.7)	2 (0.7)	

Abbreviation: *HBsAg* Hepatitis B surface antigen, *ABLI grade* albumin to bilirubin grade, *ALT* anlanine transaminase, *NLR* neutrophil to lymphocyte ratio, *PLR* platelet to lymphocyte ratio, *APRI* alanine transaminase to platelet ratio index, *PVTT* portal vein tumor thrombus, *HVTT* hepatic vein tumor thrombus, *MVI* microscopic vascular invasion, *AFP* alpha fetoprotein

### Survival impact of PBT on different stages of HCC in the entire cohort

**Fig. 1:**
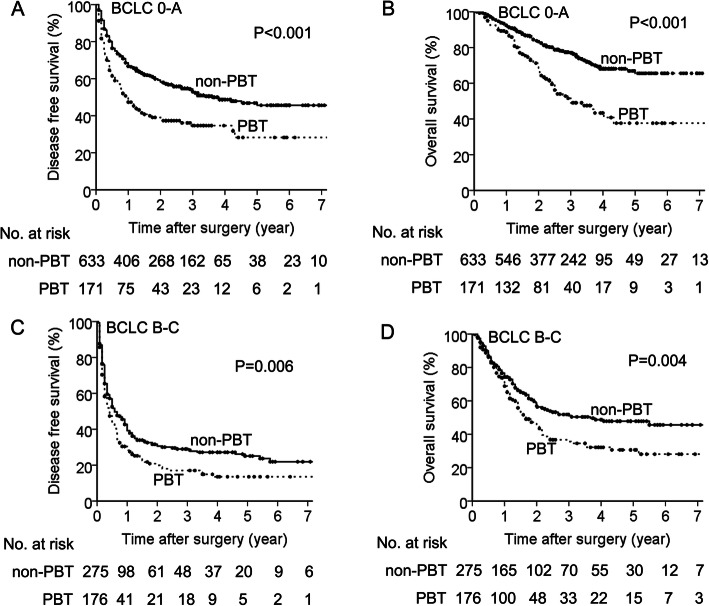
Survival curves of patients with PBT and without PBT in the BCLC 0-A subset and the BCLC B-C subset in the entire cohort. **a** DFS in the BCLC 0-A subset. **b** OS in the BCLC 0-A subset. **c** DFS in the BCLC B-C subset. **d** OS in the BCLC B-C subset (Log rank test)

In the subset of BCLC 0-A, the median DFS was 12.0 months (95% confidence interval [CI]: 7.9–16.1) for PBT group and 43.1 months (95% CI: 28.5–57.8) for the non-PBT group (*P* < 0.001) (Fig. 1a). The median OS was 36.0 months (95%CI: 25.0–47.0) for the PBT group and 96.0 months for the non-PBT group (*P* < 0.001) (Fig. 1b). In the subset of BCLC B-C, the median DFS was 5.0 months (95% CI: 3.8–6.2) for PBT group and 7.0 months (95% CI: 4.6–9.4) for the non-PBT group (*P* = 0.006) (Fig. 1c). The median OS was 20.0 months (95%CI: 14.5–25.5) for the PBT group and 44.0 months for the non-PBT group (*P* = 0.004) (Fig. 1d).

### Propensity score matching analysis

Because numerous variables were different between the PBT group and the non-PBT group in each subset of patients, a large patient selection bias existed for the entire cohort. To overcome this selection bias, PSM was used. Twenty-four variables, including patient and underlying liver disease-related, tumor-related, systemic inflammation-related, and procedure-related factors were selected as the matched factors and entered in the PSM model. After matching, 288 pairs of patients were generated from those with PBT and without PBT. In the matched cohort, apart from blood loss, the confounding variables of the matched groups in each subset were similar (Table 2). The postsurgical complication rates were comparable between patients with PBT and those without PBT within the BCLC 0-A subset and the BCLC B-C subset, respectively.

**Table 2:** Baseline characteristics of patients with PBT and those without PBT in different HCC stage subset in the matched cohort (*n* = 576)

*Variable*	BCLC 0-A (*n* = 317)			BCLC B-C (*n* = 259)		
	PBT *n* = 156)	Non-PBT (*n* = 161)	*P*-value	PBT (*n* = 132)	Non-PBT (*n* = 127)	*P*-value
*Demographic factors*						
Age, yr	52.7 ± 12.3	53.1 ± 12.4	0.787	49.2 ± 12.6	51.4 ± 13.1	0.182
Sex, n (%)						
Male	127 (81.4)	126 (78.3)	0.487	118 (89.4)	115 (90.6)	0.758
Female	29 (18.6)	35 (21.7)		14 (10.6)	12 (9.4)	
HBsAg, n (%)						
Positive	132 (84.6)	138 (85.7)	0.784	112 (84.8)	103 (81.1)	0.424
Negative	24 (15.4)	23 (14.3)		20 (15.2)	24 (18.9)	
Cirrhosis, n (%)						
Yes	144 (73.1)	121 (75.2)	0.674	101 (76.5)	93 (73.2)	0.544
No	42 (26.9)	40 (24.8)		31 (23.5)	34 (26.8)	
Hemoglobin, g/L	132.0 ± 21.5	133.4 ± 19.8	0.548	136.3 ± 21.1	133.4 ± 19.4	0.245
Platelet count, ×10^9^ /L	201.8 ± 93.3	180.6 ± 71.3	0.023	197.8 ± 85.0	207.9 ± 73.4	0.307
Prothrombin time, s	12.9 ± 1.6	12.9 ± 0.1	0.705	13.0 ± 1.1	13.0 ± 1.1	0.894
ALT, U/L, median (range)	38 (6293)	34 (71428)	0.931	44 (8, 237)	38 (12,522)	0.541
ALBI grade, n (%)						
Grade 1	82 (52.5)	77 (47.8)	0.486	64 (48.4)	53 (41.7)	0.341
Grade 2	72 (46.2)	83 (51.6)		67 (50.8)	74 (58.3)	
Grade 3	2 (1.3)	1 (0.6)		1 (0.8)	0	
*Inflammatory factors*						
NLR, median (range)	2.2 (0.52,13.03)	2.1 (0.3, 24.9)	0.904	2.3 (0.9, 24.4)	2.5 (1.1, 15.8)	0.901
PLR, median (range)	117.8 (21.4, 405.4)	107.8 (19.4, 414.0)	0.379	118.4 (19.8, 460.3)	131.1 (20.6, 314.6)	0.440
APRI, median (range)	0.5 (0.1, 5.3)	0.5 (0.1, 21.1)	0.621	0.6 (0.1, 3.8)	0.5 (0.1, 5.7)	0.269
*Tumor characteristics*						
AFP, ug/L						
≥ 400	64 (41.0)	60 (37.3)	0.493	61 (46.2)	70 (55.1)	0.153
< 400	92 (59.0)	101 (68.9)		71 (53.8)	57 (44.9)	
Tumor size, cm	8.4 ± 4.4	7.69 ± 3.86	0.110	9.8 ± 3.5	10.1 ± 3.5	0.441
Tumor number, n (%)						
Solitary	153 (98.1)	156 (96.7)	0.502	38 (28.8)	45 (35.4)	0.524
2	3 (1.9)	5 (3.1)		48 (36.4)	45 (35.4)	
3	0	0		16 (12.1)	4 (3.2)	
4	0	0		30 (22.7)	33 (26.0)	
Tumor capsule, n (%)						
Complete	127 (81.4)	135 (83.9)	0.568	86 (65.2)	81 (63.8)	0.818
Incomplete	29 (18.6)	26 (16.1)		46 (34.8)	46 (36.2)	
Differentiation, n (%)						
I+ II	108 (69.2)	111 (68.9)	0.956	88 (66.7)	85 (66.9)	0.964
III, IV	48 (30.8)	50 (16.1)		44 (33.3)	42 (33.1)	
MVI, n (%)						
Yes	35 (22.4)	33 (20.5)	0.674	62 (47.0)	66 (52.0)	0.196
No	121 (77.6)	128 (79.5)		70 (53.0)	61 (48.0)	
PVTT, n (%)						
Yes	0	0		68 (51.5)	72 (56.7)	0.405
No	156 (100)	161 (100)		64 (48.5)	55 (43.3)	
HVTT						
Yes	0	0		9 (6.8)	5 (3.9)	0.307
No	156 (100)	161 (100)		123 (93.2)	122 (96.1)	
*Surgical factors*						
Extent of resection, n (%)						
Major	59 (37.8)	58 (36.0)	0.741	91 (68.9)	83 (65.4)	0.541
Minor	97 (62.2)	103 (64.0)		41 (31.1)	44 (34.6)	
Type of resection, n (%)						
Anatomical	55 (35.3)	53 (32.9)	0.662	68 (51.5)	69 (54.3)	0.651
nonanatomical	101 (64.7)	108 (67.1)		64 (48.5)	58 (45.7)	
Resection margin, cm						
≤ 1	51 (32.7)	45 (28.0)	0.358	99 (75.0)	100 (78.7)	0.476
> 1	105 (67.3)	116 (72.0)		33 (25.0)	27 (21.3)	
Pringle maneuver, n (%)						
Yes	56 (35.9)	46 (28.6)	0.163	100 (75.7)	98 (77.2)	0.790
No	100 (64.1)	115 (71.4)		32 (24.3)	29 (22.7)	
Blood loss, ml, median (range)^a^	1000 (50, 12,000)	200 (50,3000)	< 0.001	1000 (15,7000)	300 (50,2500)	< 0.001
Clavien-Dindo grade^a^						
I	3 (1.9)	3 (1.9)	0.923	1 (0.8)	4 (3.1)	0.689
II	1 (0.6)	3 (1.9)		1 (0.8)	2 (1.6)	
III	12 (7.7)	12 (7.5)		6 (4.5)	7 (5.5)	
IV	2 (1.3)	1 (0.6)		2 (1.5)	1 (0.8)	

^a^Variables are not included in the matching model

Abbreviation: *ABLI grade* albumin to bilirubin grade, *ALT* anlanine transaminase, *NLR* neutrophil to lymphocyte ratio, *PLR* platelet to lymphocyte ratio, *APRI* alanine transaminase to platelet ratio index, *PVTT* portal vein tumor thrombus, *HVTT* hepatic vein tumor thrombus, *MVI* microscopic vascular invasion, *AFP* alpha fetoprotein, *PLR* platelet to lymphocyte ratio

### Survival impact of PBT on different stage of HCC in the matched cohort

**Fig. 2:**
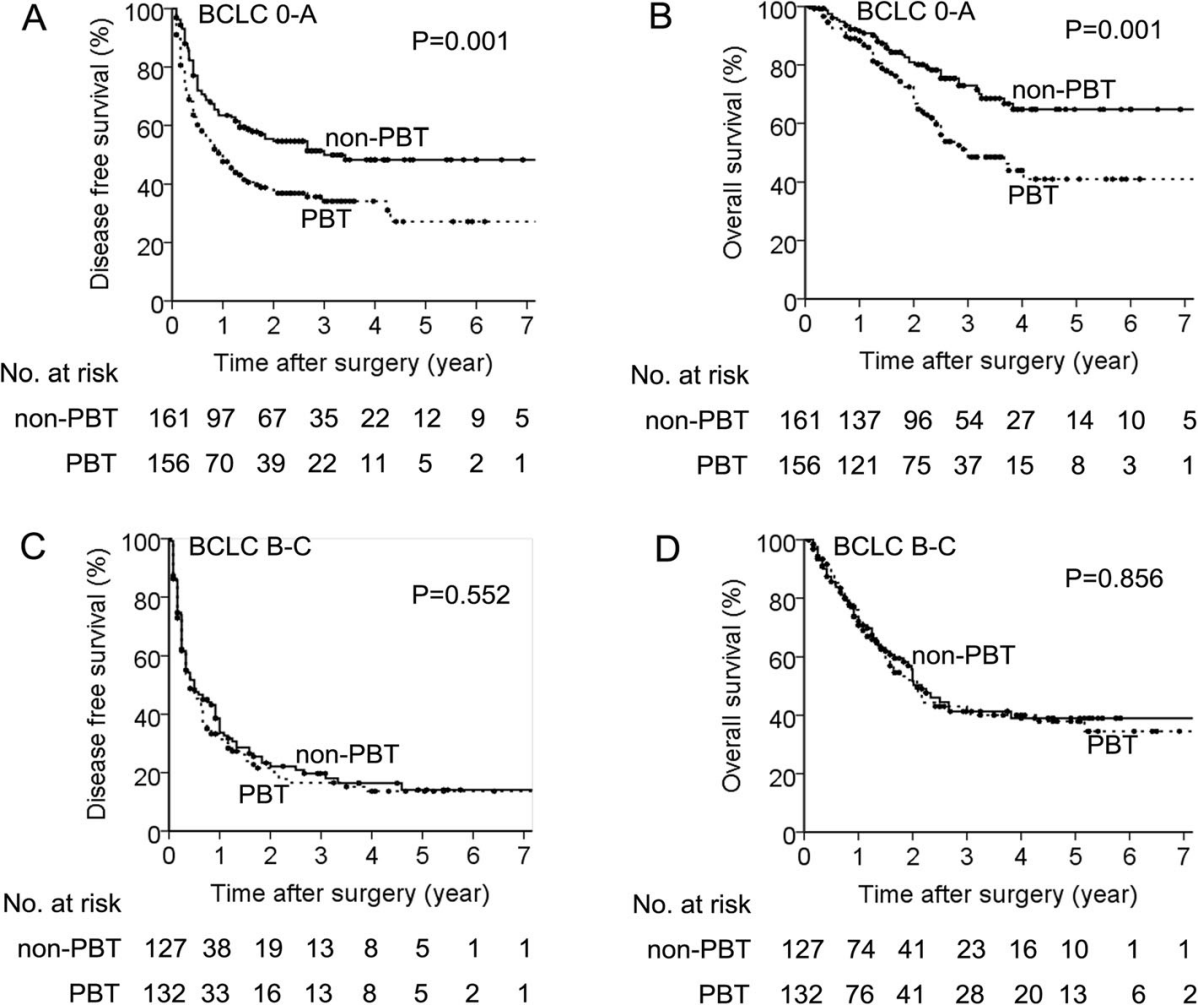
Survival curves of patients with PBT and without PBT in the BCLC 0-A subset and the BCLC B-C subset in the matched cohort. **a** DFS in the BCLC 0-A subset. **b** OS in the BCLC 0-A subset. **c** DFS in the BCLC B-C subset. **d** OS in the BCLC B-C subset (Log rank test)

There were 317 (55.0%) patients with BCLC stage 0-A, and 259 (45.0%) BCLC stage B-C in the matched cohort (Table 2). The median DFS of patients with PBT was significantly shorter than that without PBT in the BCLC stage 0-A subset (12.0 months [95%CI, 7.4–16.6] vs. 36.0 months [95% CI: 10.6–61.4], *P* = 0.001, Fig. 2a). Similar result was observed for OS (36.0 months [95% CI, 23.9–48.0] vs. 96.0 months [95% CI: 14.6–177.4], *P* = 0.001, Fig. 2b). However, the median DFS and median OS were comparable between patients with PBT and those without PBT in the subset of BCLC stage B-C HCC (both *P* > 0.05, Fig. 2c, d).

### Risk factors affecting DFS and OS

To further investigate the role of PBT in survival outcomes of HCC, the Cox regression model was used to identify the risk factors associated with DFS and OS of the entire cohort. Twenty-three clinicopathologic variables were included in the univariable analysis (Table 3). The variables with statistical significance (*P* < 0.05) were selected and entered the multivariable analysis (Table 4). The results revealed that PBT had an increased risk of DFS (hazard ratio [HR], 1.607; 95% CI,1.272–2.031; *P* < 0.001) and OS (HR, 1.756, 95% CI,1.302–2.368; *P* < 0.001) for patients with stage 0-A HCC after curative resection. However, PBT was not a risk factor of DFS or OS for patients with stage B-C HCC (both *P* > 0.05).

**Table 3:** Risk factors associated with postoperative disease free survival and overall survival identified by univariate Cox regression analysis in the entire cohort

Variables	Univariate Analysis				
	Overall survival		Disease-free survival		
	Hazard ratio	***p-valule***	Hazard ratio		***p-value***
**Age, year**					
≤ 50 vs > 50	0.991 (0.983–0.999)	0.025	0.715	(0.617–0.829)	< 0.001
**Sex**					
Male vs female	0.953 (0.710–1.278)	0.746	0.789	(0.622–0.999)	0.049
**HbsAg**					
Positive vs negative	1.013 (0.777–1.320)	0.924	1.049	(0.855–1.288)	0.646
**Cirrhosis**					
Yes vs no	1.230 (0.990–1.529)	0.062	1.325	(1.119–1.568)	0.001
**ALBI grade**					
2 + 3 vs 1	1.561 (1.291–1.886)	< 0.0001	1.218	(1.052–1.409)	0.008
**ALT, U/L**					
> 40 vs ≤ 40	1.199 (0.987–1.457)	0.068	1.254	(1.082–1.455)	0.003
**Platelet. ×10**^**9**^**/L**					
≤ 100 vs > 100	1.007 (0.690–1.469)	0.972	1.083**(**0.810–1.448**)**		0.590
**NLR**					
> 2.3 vs ≤ 2.3	1.102 (1.070–1.134)	< 0.001	1.058**(**1.030–1.087**)**		< 0.001
**PLR**					
> 118.9 vs ≤ 118.9	1.002 (1.000–1.003)	< 0.001	1.002	(1.001–1.002)	< 0.001
**APRI**					
> 0.55 vs ≤ 0.55	0.934 (0.724–1.206)	0.601	1.001	(0.847–1.184)	0.988
**Tumor size, cm**					
> 5.0 vs ≤ 5.0	2.353 (1.792–3.090)	< 0.001	1.832**(**1.523–2.204**)**		< 0.001
**Tumor number**					
Multiple vs solitary	1.906 (1.554–2.336)	< 0.001	1.768	(1.432–2.183)	< 0.001
**Tumor capsule**					
Incomplete vs complete	0.474 (0.382–0.588)	< 0.001	0.587	(0.494–0.698)	< 0.001
**Differentiation**					
3 + 4 vs 1 + 2	1.170 (0.948–1.443)	0.145	1.183	(1.007–1.389)	0.041
**Macro-VTT**					
Yes vs no	3.295 (2.660–4.083)	< 0.001	2.411	(2.026–2.869)	< 0.001
**MVI**					
Yes vs no	2.347 (1.925–2.860)	< 0.001	1.944	(1.664–2.270)	< 0.001
**AFP, μg/L**					
> 400 vs ≤ 400	1.841(1.516–2.236)	< 0.001	1.608	(1.386–1.864)	< 0.001
R**esection margin, cm**					
≤ 1.0 vs > 1.0	1.050 (0.998–1.521)	0.354	0.865 (0.775–1.211)	0.746	
**Pringle maneuver**					
Yes vs no	0.886 (0.815–0.956)	0.234	0.786 (0.705–0.898)	0.846	
**Resection type**					
Anatomic vs nonanatomic	1.366 (1.125–1.660)	0.02	1.375 (1.185–1.595)	< 0.001	
**Resection extent**					
Major vs minor	1.728 (1.422–2.099)	< 0.001	1.702 (1.468–1.974)	< 0.001	
**Blood loss, ml**					
> 800 **vs** ≤ 800	2.217(1.807–2.720)	< 0.001	1.761(1.494–2.075)	< 0.001	
**Blood transfusion**					
Yes vs no	2.107 (1.726–2.571)	< 0.001	1.759 (1.503–2.058)	< 0.001	

Abbreviation: *HBsAg* hepatitis B virus surface antigen, *ALT* anlanine transaminase, *NLR* indicates neutrophil to lymphocyte ratio, *PLR* platelet to lymphocyte ratio, *APRI* alanine transaminase to platelet ratio index, *AFP* alpha fetoprotein, *MVI* microscopic vascular invasion, *Macro-VTT* macroscopic venous tumor thrombus, including portal vein tumor thrombus and hepatic vein tumor thrombus

**Table 4:** Risk factors associated with postoperative disease free survival and overall survival identified by multivariate Cox regression analysis

Variables	OS		DFS	
	HR (95% CI)	*p*-value	HR (95% CI)	*p*-value
*The entire cohort (n = 1255)*				
Age, yr, ≤50 vs > 50			0.800 (0.688–0.931)	0.004
Cirrhosis, yes vs no			1.328 (1.117–1.578)	0.001
ALBI grade, 2 + 3 vs 1	1.225 (1.005–1.494)	0.044		
NLR, > 2.3 vs ≤ 2.3	1.080 (1.041–1.121)	< 0.001	1.034 (1.002–1.068)	0.040
Tumor size, cm, > 5 vs ≤ 5	1.437 (1.077–1.916)	0.014	1.311 (1.073–1.602)	0.008
Tumor no., multiple vs solitary	1.489 (1.206–1.838)	< 0.001	1.583 (1.343–1.866)	< 0.001
Macro-VTT, yes vs no	1.662 (1.288–2.143)	< 0.001	1.377 (1.126–1.685)	0.002
MVI, yes vs no	1.581 (1.262–1.980)	< 0.001	1.541 (1.298–1.829)	< 0.001
AFP, μg/L, > 400 vs ≤ 400	1.412 (1.154–1.726)	0.001	1.267 (1.086–1.477)	0.003
PBT, yes vs no	1.623 (1.312–2.008)	< 0.001	1.365 (1.158–1.608)	< 0.001
*BCLC 0-A subgroup (n = 804)*				
Age, yr, ≤50 vs > 50			0.986 (0.977–0.994)	0.001
Cirrhosis, yes vs no			1.325 (1.053–1.668)	0.016
ALBI grade, 2 + 3 vs 1	1.434 (1.094–1.879)	0.009		
NLR, > 2.3 vs ≤ 2.3	1.105 (1.056–1.157)	< 0.001	1.054 (1.010–1.099)	0.016
MVI, yes vs no	1.643 (1.217–2.220)	0.001	1.578 (1.252–1.988)	< 0.001
AFP, μg/L, > 400 vs ≤ 400	1.832 (1.390–2.413)	< 0.001	1.445 (1.167–1.789)	0.001
PBT, yes vs no	1.756 (1.302–2.368)	< 0.001	1.607 (1.272–2.031)	< 0.001
*BCLC B-C subgroup (n = 451)*				
Age, yr, ≤50 vs > 50			0.989 (0.980–0.999)	0.025
Tumor size, cm, > 5 vs ≤ 5			1.826 (1.151–2.897)	0.011
Tumor no., multiple vs solitary	1.546 (1.129–2.116)	0.007		
PLR, > 118.9 vs ≤ 118.9	1.002 (1.000–1.003)	0.013		
MVI, yes vs no	1.492 (1.059–2.102)	0.022	1.568 (1.253–1.961)	< 0.001
Macro-VTT, yes vs no	2.033 (1.411–2.929)	< 0.001	1.367 (1.067–1.752)	0.011
Cirrhosis, yes vs no			1.408 (1.083–1.830)	0.014
PBT, yes vs no	1.257 (0.929–1.700)	0.139	1.203 (0.950–1.525)	0.126

Abbreviation: *OS* overall survival, *DFS* disease free survival, *HR* hazard ratio, *95% CI* 95% confident interval, *ABLI grade* albumin to bilirubin grade, *NLR* neutrophil to lymphocyte ratio, *Macro-VTT* macroscopic venous tumor thrombus, *MVI* microscopic vascular invasion, *AFP* alpha fetoprotein, *PBT* perioperative blood transfusion, *PLR* platelet to lymphocyte ratio

## Discussion

The impact of PBT on survival outcomes for HCC has been debated for more than two decades [7–12, 20–24]. Because an RCT is impossible on the issue of blood transfusions in clinical practice, all evidences available were based on retrospective study. In 2013, one meta-analysis involved 22 retrospective studies with 5635 patients concluded that PBT had a negative effect on oncologic outcomes for HCC after resection [12]. However, five studies published recently still yielded controversial conclusions, although they all deliberately used a PSM analysis to adjust patient selection bias [7–11].

Resectable HCC comprised of different stage of disease, from BCLC stage 0 to C, which had large heterogeneity among patients and tumors. The prominent independent risk factors associated with recurrence or OS should be various for different stage of tumor. In the present study, we focused on the impact of PBT on HCC patient with different tumor stage and demonstrated that both DFS and OS for patients with PBT were significantly worse than those without PBT either within the BCLC 0-A subset (Fig. 1a, b) or the BCLC B-C subset (Fig. 1c, d) in the entire cohort.

Because the baseline variables of the PBT and non-PBT group within the BCLC 0-A subset or the BCLC B-C subset were diverse, patient selection bias largely existed. The patients with PBT had larger tumor burden (i.e., large tumors, multiple tumors, incomplete tumor capsules, PVTT, MVI, high levels of AFP) and higher level of inflammatory indexes (NLR, PLR and APRI) compared with those with non-PBT (Table 1). These are all well-known risk factors associated with tumor recurrence and reduced survival [25–31], as partially confirmed by the present Cox regression analysis (Table 4). This probably explains why the outcomes were worse for the patients with PBT than for those without PBT in the entire cohort.

Therefore, to overcome patient selection bias, PSM that could mimic an RCT study [32] was used. Considering that HCC recurrence is induced cooperatively by tumor-related, underlying liver disease-related, systemic inflammation-related, and procedure-related factors, the matched variables in the PSM model should comprehensively include these four outcome-related aspects to reduce selection bias as much as possible. Inclusion of more outcome-related variables in PSM would potentially reduce selection bias [33, 34]. Notably, there were 24 variables that fully covered the four aspects of risk factors described in our PSM model. The comprehensive inclusion of matched variables would maximally reduce patient selection bias in our study.

Cirrhosis, tumor size, macroscopic venous tumor thrombus and intraopeative blood loss were reported to be the risk factors associated with PBT [9, 11]. Excessive blood loss is the most important cause of PBT. PBT or blood loss, which one is the prominent factor affecting oncologic outcome is clinically hard to define, although a previous study showed that blood loss predicted recurrence and poor OS [35]. In the present study, Cox univariable analysis showed that both blood loss and PBT were significant risk factors of DFS and OS (Table 3). However, it was PBT rather than blood loss affecting both DFS and OS in multivariable analysis (Table 4). Therefore, we believed although blood loss was not adjusted as a selected factor for propensity matching, it would not potentially affect the survival results derived from the matched cohort.

After propensity matching, the baselines of patients with PBT and those without PBT were comparable (Table 2) within the BCLC 0-A subset or the BCLC B-C subset. The survival results showed that PBT significantly reduced postoperative DFS and OS of HCC patients with BCLC stage 0-A (Fig. 2a, b), but it no longer influence the postsurgical survival outcomes of those with BCLC stage B-C (Fig. 2c, d). These were consistent with an early study reported by Ashara et al. in 1999, but our study had superiority in patient number and statistical power. In that study, only 175 patients were included and PSM was not applied to control patient bias [36].

27.6% patients required blood transfusion in the entire cohort, but they all achieved curative resection (complete resection of gross tumors with a pathological tumor free margin). Therefore, the volume of intraoperative blood loss does not correlate with the curativity of resection for HCC. To further evaluate the impact of PBT on survival outcomes of HCC, Cox univariable and multivariable regression analyses were performed in the matched cohort. The results showed that PBT, but not blood loss was associated with a reduced DFS and OS (Table 4). PBT was significantly associated with increased risks of poor DFS and OS for the subset of patients with BCLC stage 0-A HCC. However, in the BCLC B-C subset, PBT was not a risk factor affecting DFS and OS. Tumor-related factors (multiple tumor, size, venous tumor thrombus, MVI) are the major risk factors associated with tumor recurrence and OS. In the subset with early tumor, patients with PBT had a shorter DFS or OS may partially result from transfusion-related immunomodulation (TRIM) [37]. Residual leukocyte or apoptotic cells in the stored RBCs may stimulate TGFβ and TNFα production, which in turn suppresses NK cells and activate Treg cells. Furthermore, microparticles derived from RBCs may contribute to neutrophil priming and activation and promotion of inflammation. These collectively caused immunosuppression [38], thereby promoting tumor recurrence.

This study had several limitations. First, it was a retrospective cohort study, not an RCT trial. However, the large sample size and the combination of PSM (full inclusion of variables and appropriate calipers) and Cox regression analyses strengthened the statistical data, thereby yielding reliable results. Second, it was a single-center study, and most patients had hepatitis B virus-related HCC. External validation by other independent cohorts with different HCC etiologies is needed.

## Conclusions

The present study demonstrated that PBT would significantly reduce DFS and OS of patients with BCLC stage 0-A HCC, but not those of patients with BCLC stage B-C HCC after curative liver resection. Deliberate preoperative planning and refined intraoperative manipulation are required to minimize blood loss and transfusion, thereby improving outcomes of HCC.

## Data Availability

All data generated or analysed during this study are included in this published article.

## Abbreviations

AFP: Alpha fetal protein
ALBI: Albumin to bilirubin
APRI: Alanine transaminase -to-platelet ratio index
ALT: Alanine transaminase
AR: Anatomical resection
BCLC: Barcelona Clinic Liver Cancer
95% CI: 95% confidence interval
DFS: Disease free survival
HCC: Hepatocellular carcinoma
HR: Hazard ratio
HVTT: Hepatic vein tumor thrombus
MVI: Microscopic vascular invasion
NAR: Non-anatomical resection
NLR: Neutrophil to lymphocyte ratio
OS: Overall survival
PBT: Perioperative blood transfusion
PLR: Platelet to lymphocyte ratio
PSM: Propensity score matching
PVTT: Portal vein tumor thrombus
